# Calorie and time-restricted feeding improves liver and kidney histopathology in streptozotocin-induced type 1 diabetic rats

**DOI:** 10.3389/fphys.2025.1629751

**Published:** 2025-08-21

**Authors:** Indra Putra Taufani, Sri Rahmatul Laila, Sri Tasminatun, Sailent Rizki Sari Simaremare, Meity Mardiana, Jiro Hasegawa Situmorang

**Affiliations:** ^1^ Department of Pharmacist Profession Education, Faculty of Medicine and Health Sciences, Universitas Muhammadiyah Yogyakarta, Yogyakarta, Indonesia; ^2^ Division of Anatomy, Histology, and Embryology, School of Veterinary Medicine and Biomedical Science, IPB University, Bogor, Indonesia; ^3^ School of Pharmacy, Faculty of Medicine and Health Sciences, Universitas Muhammadiyah Yogyakarta, Yogyakarta, Indonesia; ^4^ Research Center for Public Health and Nutrition, National Research and Innovation Agency (BRIN), Cibinong, Indonesia; ^5^ Biotechnology Department, Indonesia International Institute for Life Sciences (i3L), Jakarta, Indonesia; ^6^ Center for Biomedical Research, National Research and Innovation Agency (BRIN), Cibinong, Indonesia

**Keywords:** type 1 diabetes, calorie restriction, time-restricted feeding, metabolic dysfunction, circadian rhythm, hepatomegaly, diabetic nephropathy, glucose tolerance

## Abstract

Type 1 diabetes (T1D) is associated with severe metabolic dysregulation and organ complications such as hepatomegaly and nephropathy. While insulin therapy remains the cornerstone of treatment, there is growing interest in dietary interventions that modulate metabolic outcomes independently of insulin. This study aimed to investigate the effects of calorie restriction (CR) combined with time-restricted feeding (TRF) on metabolic and histological parameters in a high-fat diet-fed, streptozotocin-induced rat model of T1D. Male Sprague-Dawley rats were divided into control, diabetic, and two CR-TRF groups (day-fed and night-fed). CR-TRF groups received 70% of the diabetic group’s caloric intake during either the light or dark phase. Body weight, fasting glucose, oral glucose tolerance test (OGTT), triglycerides, water intake, and calorie intake were measured. Liver and kidney tissues were evaluated using H&E and Cason’s Trichrome staining. Although CR-TRF did not significantly improve body weight, both interventions markedly reduced water intake and improved hepatomegaly. OGTT results showed improved slight glycemic responses in CR-TRF groups, particularly in the day-fed group. Diabetic rats exhibited liver and renal damage, which were significantly attenuated by CR-TRF. Histological analysis revealed preserved tissue architecture and reduced vacuolation in both liver and kidney under CR-TRF conditions. These findings support the potential of calorie restriction, regardless of feeding time as adjunct therapies for T1D and warrant further exploration in translational models.

## 1 Introduction

Type 1 diabetes (T1D) affects approximately 8.4 million people worldwide, and this number is projected to increase significantly by 2040 (Gregory et al., 2022). This chronic autoimmune disorder is characterized by the progressive destruction of insulin-producing beta cells in the pancreas, leading to lifelong insulin dependence. The clinical manifestations of T1D such as hyperglycemia, ketoacidosis, polyuria, and polydipsia significantly affect the quality of life of individuals living with the disease. Despite advances in insulin therapy and management strategies, the ability to reverse or cure T1D remains elusive, necessitating the development of novel therapeutic interventions to mitigate disease progression and improve patient outcomes.

Calorie restriction (CR) and time-restricted feeding (TRF) have shown beneficial effects in several metabolic disorders, especially in obesity and Type 2 diabetes (T2D). CR improves insulin sensitivity and reduces adiposity (Dos Santos et al., 2024) (Johnson et al., 2015), and prevent the development of complication associated with T2D, such as nephropathy (Ruggenenti et al., 2022). On the other hand, TRF, a dietary strategy that confines food intake to a specific window during the day, offers a novel approach to improving metabolic health. Typically, TRF involves fasting for extended periods such as 12–18 h while eating during a restricted feeding window of 6–12 h. This strategy aligns the body’s circadian rhythms, which regulate various metabolic processes, including glucose metabolism, insulin sensitivity, and cellular repair mechanisms (Manoogian et al., 2022). Even with *ad libitum* high-fat diet (HFD), TRF alone has been shown to protect against metabolic disturbances such as obesity, hyperinsulinemia, hepatic steatosis, and inflammation (Hatori et al., 2012).

Study showed that CR and TRF combination can extend lifespan, with mice fed during the daytime only showing a 20% lifespan extension, while those fed exclusively at night demonstrated a more significant increase in lifespan of 35% (Acosta-Rodriguez et al., 2022). This significant contrast, despite both groups of mice consuming similar calories, indicates that circadian rhythms play a crucial role in long-term health outcomes and overall body metabolism.

While several studies have documented the efficacy of CR and TRF in obesity and T2D models, limited research exists on their roles in T1D, particularly in non-insulin-treated models. Most T1D research has focused on pharmacological therapies, but emerging studies suggest that diet-based interventions may influence disease course through non-insulin-dependent pathways (Kahleova et al., 2024). Moreover, the impact of feeding timing particularly day vs. night feeding under CR has not been evaluated in T1D contexts.

This study aimed to assess the combined effects of CR and TRF on metabolic parameters and organ histopathology in a high-fat diet-fed, streptozotocin-induced T1D rat model. We compared outcomes between rats fed during the day or night under calorie restriction. We hypothesized that the integration of TRF with CR would alleviate T1D complications such as hyperglycemia and that feeding timing could influence the degree of improvement.

## 2 Materials and methods

### 2.1 Materials

Eosin (318906) and Glucose (G7021) were purchased from Sigma-Aldrich (St. Louis, MO, United States). The Mason trichrome stain kit (ab150686) was purchased from Abcam (Cambridge, United Kingdom). Hematoxylin (H3401) was purchased from Vector Laboratories (Newark, CA, United States). The High-Fat Diet (D12492) was purchased from Research Diets (NJ, United States). Glucose strips (ACCU-CHEK) were purchased from Roche (Indianapolis, IN, United States). Strips for TG measurement (LipidPro) were purchased from OSANG Healthcare (Korea).

### 2.2 Animal experiment

Animal experiments were carried out in strict accordance with the National Institutes of Health Guide for Care and Use of Laboratory Animals and with the approval of the Health Research Ethics Committee (approval no: 094/KE.03/SK/04/2024) of National Research and Innovation Agency (BRIN), Indonesia. Furthermore, all procedures conducted in the animal study were in accordance with the ARRIVE guidelines.

Eight-week-old male Sprague-dawley rats were purchased from Indonesian Food and Drug Authority. Upon arrival, the rats were acclimated for 1 week and housed in a controlled environment with a temperature of 25°C ± 1°C and humidity levels of 55%–60%, maintained under a 12-h light/dark cycle. Afterward, the rats were randomly divided into four groups: the control group, which received an *ad libitum* chow diet; the diabetes group, which received an *ad libitum* high-fat diet; the diabetes day group, which received a calorie-restricted high-fat diet from 6 a.m. to 6 p.m.; and the diabetes night group, which received a calorie-restricted high-fat diet from 6 p.m. to 6 a.m., with food administered at 2-h intervals. In both calorie-restricted groups, the daily food intake was limited to 70% of the amount consumed by the *ad libitum* diabetes group.

Before inducing diabetes with streptozotocin (STZ) (40 mg/kg) (Furman, 2021), all three diabetes groups were given an *ad libitum* high-fat diet for 28 days. One week after STZ induction, fasting blood glucose levels were measured, and rats with fasting glucose levels above 200 mg/dL were included in the study. Calorie and time-restricted feeding were then initiated according to the assigned groups.

### 2.3 Triglyceride, fasting blood glucose and oral glucose tolerance measurement

For triglyceride and fasting blood glucose measurement, rats were fasted for 6 h prior to assessment. For the oral glucose tolerance test, rats were fasted for 12 h, after which 1.5 g/kg of glucose in a 20 mL/kg sodium chloride solution was administered via intragastric feeding. Triglyceride and blood glucose levels were then measured using strip-based analysis, with blood samples collected from the tip of the rat’s tail.

### 2.4 Histological staining of the liver

Liver and kidney tissues were fixed in 10% formalin, embedded in paraffin, sectioned at 4–5 μm thickness, and stained with hematoxylin and eosin (H&E) or Cason’s Trichrome (CT) using standard protocols. For H&E staining, tissues were stained with hematoxylin for 5 min, rinsed, r running tap water. Slides were then stained with Casand counterstained with eosin for 2 min. For CT staining, nuclear staining was performed with Weigert’s iron hematoxylin for 5 min, followed by a 2-min rinse undeson’s trichrome for 5 min and briefly rinsed. All sections were subsequently dehydrated through a graded alcohol series, cleared in xylene, and mounted with coverslips.

### 2.5 Statistical analysis

The data were presented as mean ± S.E.M. and were statistically analyzed and graphed using GraphPad Prism. All data were assumed to follow a normal distribution. One-way ANOVA and *post hoc* Dunnett multiple comparisons were conducted. A p-value of <0.05 was regarded as statistically significant.

## 3 Results

### 3.1 Effect of CR-TRF on body weight, water consumption, and liver weight

**FIGURE 1:**
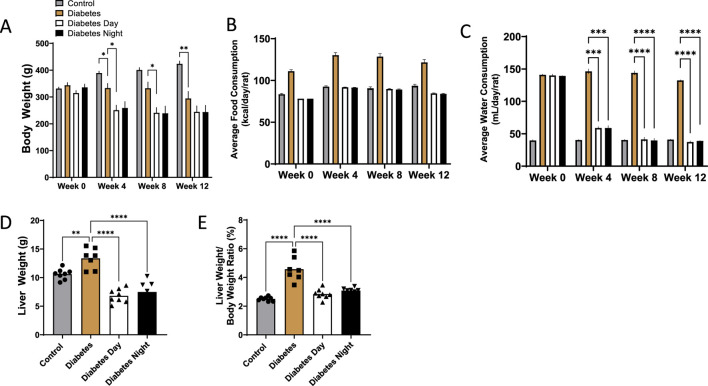
**(A)** Weekly progression of body weight over 12 weeks. **(B)** Average daily food consumption at weeks 0, 4, 8, and 12. **(C)** Average daily water consumption at weeks 0, 4, 8, and 12. **(D)** Liver weight at endpoint. **(E)** Liver-to-body weight ratio at endpoint. Data are presented as mean ± SEM (n = 7-8 per group). Statistical analysis was performed using one-way ANOVA with Dunnett’s *post hoc* test. **p < 0.01, ***p < 0.001, ****p < 0.0001 vs. diabetic group.

Throughout the 12-week intervention period, body weight increased progressively in the control group. All diabetic rats exhibited significantly lower body weight compared to controls. However, no significant differences were observed between the diabetic and CR-TRF groups by week 12 (Figure 1A). Over time, diabetic rats without CR-TRF intervention exhibited higher daily calorie consumption, whereas diabetic rats undergoing CR-TRF consumed calories at levels comparable to the control group (Figure 1B). Water consumption was markedly elevated in the diabetic group, indicating polydipsia. Both the daytime and nighttime CR-TRF groups showed significantly reduced water intake starting from week 4, reaching levels comparable to the control group by week 12 (Figure 1C). Liver weight (Figure 1D) and liver-to-body weight ratio (Figure 1E) were significantly elevated in diabetic rats, indicating hepatomegaly. These parameters were substantially reduced in the CR-TRF groups, suggesting that the dietary intervention effectively prevented liver enlargement.

### 3.2 Effect of CR-TRF on fasting glucose, glucose tolerance, and triglyceride levels

**FIGURE 2:**
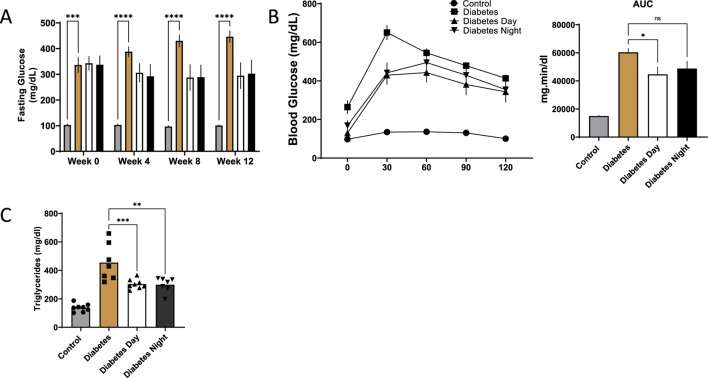
**(A)** Fasting blood glucose levels measured at weeks 0, 4, 8, and 12. **(B)** Oral glucose tolerance test (OGTT) performed at week 12 and area under the curve (AUC) analysis of OGTT. **(C)** Plasma triglyceride levels at week 12. Data are shown as mean ± SEM (n = 7-8 per group). Statistical comparisons were conducted using one-way ANOVA followed by Dunnett’s test. *p < 0.05, **p < 0.01, ***p < 0.001, ****p < 0.0001 vs. diabetic group.

Fasting blood glucose levels were significantly elevated in diabetic rats compared to the control group at all time points. CR-TRF treatment resulted in a gradual reduction in fasting glucose levels over the 12 weeks. Although the decreases in both CR-TRF groups were not statistically significant compared to the untreated diabetic group, fasting glucose values appeared consistently lower by week 12 (Figure 2A). During the OGTT at week 12, diabetic rats showed a sharp rise in blood glucose levels post-glucose load. While glucose tolerance remained impaired in all diabetic groups, the daytime CR-TRF group demonstrated a trend toward better glycemic control compared to diabetic group, with significantly lower glucose levels at 30-min and a reduced area under the curve (AUC) in daytime feeding group (Figure 2B). Triglyceride levels at week 12 were also significantly elevated in diabetic rats, and both CR-TRF treatment partially reduced these levels (Figure 2C).

### 3.3 Histopathological improvement in liver and kidney tissues following CR-TRF feeding in diabetes

**FIGURE 3:**
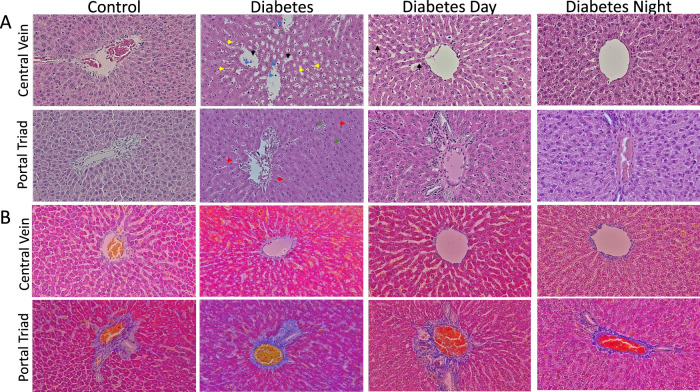
**(A)** Representative liver sections stained with hematoxylin and eosin (H&E) at 40X magnification showing hepatic architecture. Control livers exhibit normal morphology with minimal vacuolation. Diabetic livers display sinusoidal dilation (black arrow), inflammatory infiltration (blue arrow), necrosis (yellow arrow), microvesicular steatosis (red arrow), and karyomegaly (green arrow). CR-TRF groups show preserved liver architecture and reduced pathological changes compared to diabetic controls. **(B)** Representative liver sections stained with Casson’s Trichrome (CT) at 40X magnification show no clear indication of fibrosis in any group.

The histological analysis of liver sections stained with H&E (Figure 3A) revealed several notable findings. The control liver displayed normal hepatic cords radiating from central veins and portal triad, with hepatocytes exhibiting uniform morphology and minimal cytoplasmic vacuolation. Sinusoidal spaces were clear and well-defined, showing no signs of inflammatory infiltration or fibrosis. In contrast, the diabetic liver demonstrated increased sinusoidal dilation, microvesicular steatosis, and notable inflammatory cell infiltration. Hepatocytes exhibited features of cellular stress, including nuclear enlargement (karyomegaly) and necrosis. In the CR-TRF treatment groups, hepatic architecture was largely preserved, characterized by reduced sinusoidal dilation, diminished inflammatory infiltration, and fewer signs of cellular stress. Compared to the diabetic liver, CR-TRF-treated livers showed fewer degenerative changes, less steatosis, and improved overall structural integrity. However, CT staining results (Figure 3B) show no clear indication of fibrosis, suggesting that by week 12, our diabetic model had not developed detectable fibrotic changes.

**FIGURE 4:**
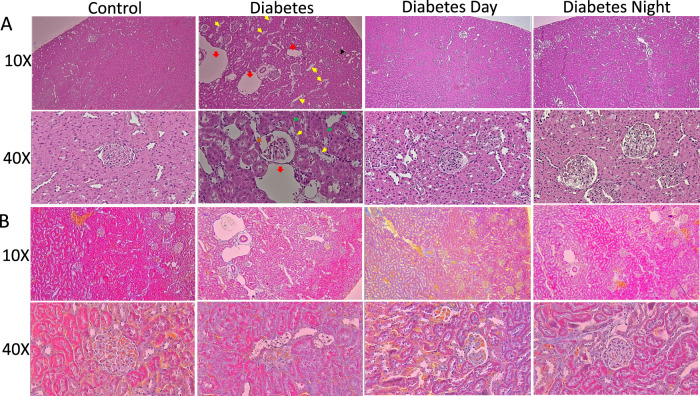
**(A)** Representative sections of the kidney cortex stained with H&E at 10X and 40X magnification. Control kidneys show normal glomerular and tubular structures. Diabetic kidneys demonstrate congestion (black arrow), tubular dilation (red arrow), tubular degeneration (yellow arrow), granular cast (green arrow) and mild Bowman’s space G/B ratio (brown arrow). CR-TRF treatment partially preserves kidney morphology with reduced lesion severity. **(B)** Representative kidney sections stained with Casson’s Trichrome (CT) at 10X and 40X magnification show no clear indication of fibrosis in any group.

For the kidney, the histological analysis from H&E (Figure 4A) shows normal glomeruli with well-defined capillary loops in the control rats. The renal tubules were arranged regularly, with clear cytoplasm, intact brush borders, and no vacuolation or signs of dilation or degeneration. Tubular epithelial cells appeared normal, with no significant cytoplasmic vacuolation or nuclear enlargement. The interstitial space had minimal inflammatory infiltration, with no expansion. The diabetic kidney exhibited tubular dilation and degeneration, and mild Bowman/s space G/B ratio suggested early signs of diabetic nephropathy. The CR-TRF treatment showed relatively intact glomeruli with mild vacuolation and some dilation, though less severe than in the diabetic sample. Similar to the liver, CT staining results (Figure 4B) of the kidney show no clear indication of fibrosis.

## 4 Discussion

This study evaluated the effects of combining CR and TRF on metabolic parameters and histological outcomes in STZ-induced T1D rats. Despite no significant differences in body weight between diabetic and CR-TRF groups after 12 weeks, we observed a clear improvement in water consumption and hepatomegaly in the CR-TRF groups. Hepatomegaly and polydipsia are known complications of poorly controlled T1D (Jardim et al., 2013). Water intake, which was significantly elevated in the diabetic group, was reduced to near-normal levels in both CR-TRF groups, suggesting improvement in polydipsia a classical symptom of uncontrolled diabetes.

Initially, we anticipated a substantial reduction in blood glucose levels in the CR-TRF groups. However, our results showed no statistically significant differences in fasting glucose at weeks 4, 8, and 12 post-interventions. Nevertheless, a downward trend in fasting glucose levels was observed in both intervention groups: from 342 mg/dL at week 0 to 294 mg/dL at week 12 in the Diabetes Day group, and from 337 mg/dL to 302 mg/dL in the diabetes Night group. In contrast, the diabetic control group showed a progressive increase, from 336 mg/dL at week 0 to 446 mg/dL at week 12. These findings suggest that although CR-TRF may not normalize glucose levels, it effectively prevents further deterioration in fasting glucose despite all groups receiving the same high-fat diet. Similarly, glucose tolerance, as assessed by OGTT, was improved in both CR-TRF groups, with statistical significance observed only in the Diabetes Day group. Interestingly, although rodents are nocturnal and feeding during the daytime is considered a form of circadian misalignment (Acosta-Rodriguez et al., 2024), the Diabetes Day group exhibited slightly better glucose control than the Diabetes Night group. This unexpected finding suggests that, in this T1DM, circadian timing of feeding may play a lesser role, while substrate restriction through reduced caloric intake and extended fasting duration appears to be the dominant factor driving the observed blood glucose control improvements.

Given that insulin was undetectable in our T1DM model, the observed partial improvement in glycemia is notable. This indicates that CR-TRF can modulate glucose homeostasis through insulin-independent mechanisms. One likely contributor is the reduction in hepatic gluconeogenesis due to limited availability of substrates such as amino acids and glycerol during calorie restriction (Sathananthan et al., 2015; She et al., 2021). Insulin-independent glucose uptake pathways, including GLUT1 and GLUT3 in the brain, muscle, and erythrocytes, may also contribute modestly to glucose clearance.

Diabetic rats showed marked hepatomegaly, as reflected by significantly increased liver weight and liver-to-body weight ratio, whereas this effect was prevented in the CR-TRF groups, where liver and body weight ratio remained closer to control levels. Histological examination of liver and kidney tissues further supported the protective role of CR-TRF, revealing reduced necrosis, sinusoidal and tubular dilatation, degeneration, and overall structural damage. Since both CR-TRF interventions resulted in comparable improvements in liver and kidney histopathology, we suggest that substrate restriction and feeding schedule rather than circadian alignment are the primary factors mediating these benefits in T1DM.

The potential mechanisms underlying the protective effects of CR-TRF on organ health and polydipsia are complex and likely involve multiple biological pathways. CR may help reduce excessive fat accumulation in organs like the liver, which becomes enlarged due to insulin deficiency and fat deposition (Aluko et al., 2020; Park et al., 2017). While the precise molecular mechanisms remain to be elucidated, possible pathways include reduced oxidative stress, improved lipid handling, and suppression of pro-inflammatory cytokines.

Limitations of this study include the absence of antidiabetic agents, insulin therapy and lack of molecular to support mechanistic insights. However, our findings provide proof-of-concept evidence that CR-TRF may be beneficial in T1D even in the absence of insulin intervention. Additionally, since early signs of diabetic nephropathy were observed, future studies should assess cardiovascular parameters, investigate the underlying molecular mechanisms, consider sex-specific responses, and evaluate whether combining CR-TRF with insulin therapy or antidiabetic agents could enhance therapeutic outcomes in T1D.

## 5 Conclusion

In summary, CR and TRF improved liver and kidney histopathology and reduced water intake in STZ-induced type 1 diabetic rats. While glucose control was only slightly improved, however at least CR-TRF appeared to prevent further deterioration in glycemic status. The prevention of hepatomegaly and polydipsia suggests that dietary timing and restriction offer protective effects against T1D complications. These findings support further investigation into CR and TRF as adjunct dietary strategies in T1D management.

## Data Availability

The raw data supporting the conclusions of this article will be made available by the authors, without undue reservation.
